# Plant colonization mediates the microbial community dynamics in glacier forelands of the Tibetan Plateau

**DOI:** 10.1002/imt2.91

**Published:** 2023-02-14

**Authors:** Yang Liu, Mukan Ji, Wenqiang Wang, Tingting Xing, Qi Yan, Belinda Ferrari, Yongqin Liu

**Affiliations:** ^1^ Center for Pan‐third Pole Environment Lanzhou University Lanzhou China; ^2^ State Key Laboratory of Tibetan Plateau Earth System, Resources and Environment (TPESRE), Institute of Tibetan Plateau Research Chinese Academy of Sciences Beijing China; ^3^ University of Chinese Academy of Sciences Beijing China; ^4^ School of Biotechnology and Biomolecular Sciences Australian Centre for Astrobiology UNSW Sydney Randwick New South Wales Australia

## Abstract

It has long been recognized that pH mediates community structure changes in glacier foreland soils. Here, we showed that pH changes resulted from plant colonization. Plant colonization reduced pH and increased soil organic carbon, which increased bacterial diversity, changed the community structure of both bacteria and fungi, enhanced environmental filtering, and improved microbial network disturbance resistance.

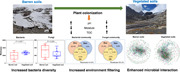

## INTRODUCTION

Global warming has increased the rate of glacier retreat in both high‐latitudes and high‐altitude regions over the past 100 years [1, 2]. Retreating glaciers expose a large mass of frequently oligotrophic sediment that has been previously locked under the ice [3, 4]. Microorganisms are the pioneer colonizers of newly exposed ground (glacier foreland) [5, 6], their community dynamics determine ecosystem functions, which ultimately influence the carbon transformation processes [7, 8, 9]. Glacial foreland microbial flora comprises bacteria, fungi, and microeukaryotes [5, 6]. Understanding their changes and their environmental determinants is essential to predict their response to climate warming and anthropogenic impact. This is particularly vital as the size of glacier foreland has been expanding rapidly over the past 100 years due to global warming and the subsequent glacier retreat [1, 2], which has turned the glacier foreland into a source of carbon dioxide and methane [10, 11, 12].

The environmental determinants of microbial communities in glacier foreland vary depending on the geographical scale of the study, the ecosystem types, and the targeted microorganisms. For example, Hailuogou 120 years deglaciation chronosequence from barren to forest soils showed that phosphorus, soil pH, and soil organic carbon (SOC) explained bacterial succession, while SOC, grazers, and pH explained fungal succession [13]. High Arctic 10 years deglaciation chronosequence revealed the importance of SOC in the bacterial community [14]; while other studies have identified the time since deglaciation as the dominant driver [15, 16, 17, 18]. Bacteria and fungi in glacier foreland have distinct community changing patterns as fungal community is more constrained by dispersal limitation due to their larger cellular sizes [5, 19, 20]. Fungal community loss diversity is opposing the increasing trend of bacteria, while other studies have observed increasing trends [21]. The different community changing patterns of bacteria and fungi during deglaciation suggests that they follow different successional trajectories [5, 14, 20, 22, 23].

Among other physicochemical factors, pH and organic carbon are most frequently identified as important environmental drivers [15, 24, 25, 26, 27, 28]. Organic carbon typically increases with time since deglaciation, which resulted from microbial autotrophs and plant inputs [4, 23, 29]. Microbial autotrophs (such as Cyanobacteria) are the primary carbon fixers before plant colonization [4, 30], while plant colonization has a much greater impact and can further increase SOC by 1.8 folds [31]. In comparison, Tripathi et al. conducted a meta‐analysis of six glacier foreland soils globally, proposing that soil pH is the key factor mediating the balance between stochastic and deterministic processes in bacterial assembly [24]. However, the ecosystem types of the six glacier forelands were not explicitly presented. In fact, if both vegetated and barren soils are included in the study, pH changes could be the consequence of plant colonization. Thus, plant colonization as a source of both pH and SOC changes could be the primary driver of microbial community dynamics in glacier foreland. This proposition has been proposed by Brown and Jumpponen that plant colonization, but not the identity of the plants, has great impact on the community dynamics of both bacterial and fungi, but has not been fully tested [23].

Most glacier foreland studies focus on a single glacier, which may explain their inconsistency on the environmental drivers and microbial successional patterns [5, 16, 25]. Analyses of geographically separated glaciers may provide additional insights into the community dynamics of microorganisms in glacier foreland. The Tibetan Plateau has the third largest number of glaciers globally, which are retreating at an unprecedented rate [1, 32]. Thus, this provides an opportunity to integrate multiple glacier forelands and investigate the foreland microbial dynamics across the Tibetan Plateau. Due to the connection between plant colonization and soil physicochemical changes, we propose that plant colonization is the fundamental driver of microbial community dynamics in glacier foreland, rather than an individual physicochemical factor. If the above assumption holds, the environmental determinants should be different before and after plant colonization.

**Figure 1:**
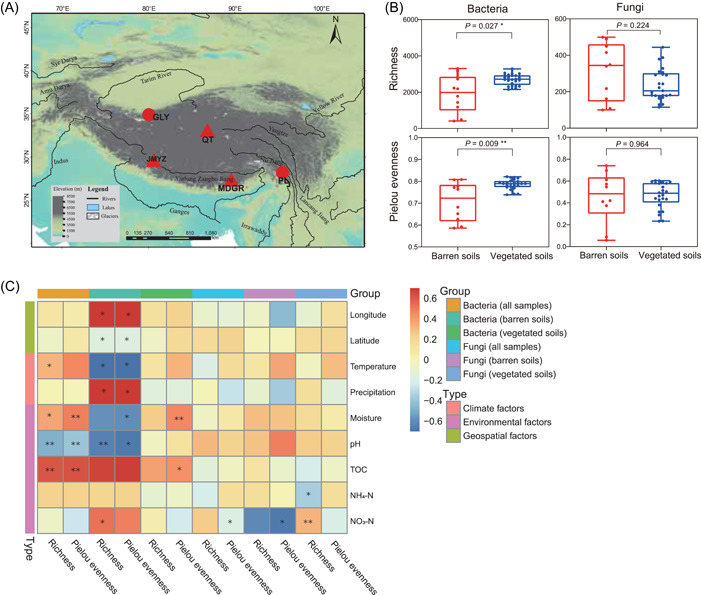
Sampling map and variations in microbial diversity. (A) The map shows the soil sampling locations in glacier foreland across the Tibetan Plateau; vegetated soils were collected near Jiemayangzong (JMYZ), Mengdagangri (MDGR), and Qiangtang No. 1 glaciers (QT); barren soils were collected near Parlung (PL) and Guliya (GLY) glaciers. The circle represents barren soils, and the triangle represents vegetated soils. (B) The influence of plant colonization on bacterial and fungal alpha‐diversity indices. (C) Correlation analysis of microbial diversity indices with environmental, geospatial, and climate factors. Asterisk indicates significant differences (***p < 0.001; **p < 0.01; *p < 0.05). TOC stands for total organic carbon.

To investigate the effect of plant colonization on community dynamics of bacterial and fungal communities in glacier forelands, we sampled soils from five geographically separated glacier forelands across the Tibetan Plateau to account for the potential influence of soil texture differences (Figure 1A). We propose that (1) the impact of pH on microbial community only occurs after plant colonization, while carbon consistently explains microbial community both before and after plant colonization; (2) as bacteria and fungi follow different successional trajectories and are explained by different soil physicochemical properties, their diversity and community structure changing patterns to plant colonization could also be different.

## RESULTS

### Changes in soil physicochemical properties

Plant colonization significantly changed the measured soil physicochemical properties across the five glacier forelands investigated. Soil moisture, total organic carbon (TOC), and NH_4_‐N were significantly higher in vegetated soils (increased by 47.5%, 2.3%, and 5.3 mg/kg, respectively; Mann–Whitney *U* test, all *p* < 0.01), while pH and soil NO_3_‐N were significantly lower (decreased by 1.1 and 28 mg/kg, respectively; Figure S1). In barren soils, soil physicochemical differences between glaciers were not compared due to the lower sample size of Guliya (GLY) glacier (*n* = 2). For vegetated soils, TOC, and NH_4_‐N concentrations were not significantly different (Figure S1C,D), while the other measured soil properties (soil moisture, pH, and NO_3_‐N) were significantly different (Table 1 and Figure S1A,B,E).

**Table 1:** Comparison of soil physicochemical properties between barren and vegetated soils.

Glaciers	Moisture (%)	pH	TOC (%)	NH_4_‐N (mg/kg)	NO_3_‐N (mg/kg)
Barren soils	11.85 ± 9.35^A^	7.71 ± 0.48^B^	1.50 ± 2.48^A^	4.60 ± 4.72^A^	40.39 ± 27.77^B^
PL	11.13 ± 10.44	7.65 ± 0.52	1.84 ± 2.69	4.69 ± 5.34	43.66 ± 30.46
GLY	14.75 ± 1.39	7.95 ± 0.04	0.14 ± 0.05	4.07 ± 0.40	27.29 ± 4.28
Vegetated soils	59.43 ± 23.17^B^	6.60 ± 0.84^A^	3.83 ± 2.58^B^	9.86 ± 7.56^B^	12.43 ± 12.46^A^
JMYZ	46.94 ± 18.22^a^	6.29 ± 0.61^a^	4.22 ± 2.35^a^	10.00 ± 11.32^a^	22.24 ± 11.32^b^
MDKR	52.47 ± 30.97^ab^	5.80 ± 0.68^a^	5.31 ± 2.94^a^	9.16 ± 3.95^a^	12.09 ± 14.50^b^
QT	74.84 ± 12.24^b^	7.37 ± 0.30^b^	2.60 ± 2.17^a^	10.15 ± 5.44^a^	3.79 ± 2.03^a^

*Note*: Capital letters indicate significant differences (at *p* < 0.05) between the barren and vegetated soils; lowercase letters indicate significant differences among glaciers in vegetated soils.

Abrreviations: GLY, Guria glacier; JMYZ, Jiemayangzong glacier; MDGR, Mengdagangri glacier; PL, Parlung glacier; QT, Qiangtang No. 1 glacier.

### Influence of environmental parameters on microbial alpha‐diversity

There were 14,473 bacterial operational taxonomic units (OTUs) identified across all samples, 2065 of which (14% of the total species pool) were specific to barren soils, 6675 (46%) were specific to vegetated soils, whereas 5733 OTUs (40%) were common to both (Figure S2A). In comparison, there were 4789 fungal OTUs identified across all samples, 1837 of which were specific to barren soils (38% of the total species pool), 2522 (53%) were specific to vegetated soils, whereas only 430 (9%) were common to both, which is less than that of bacteria (Figure S2B).

The richness and Pielou evenness of bacteria in barren soils (averaged at 1890.8 and 0.7, respectively) were significantly lower than those in vegetated soils (2702.8 and 0.8, respectively; Mann–Whitney *U* test, *p* = 0.03 and 0.009, respectively; Figure 1B). The alpha‐diversity indices were similar among the different glaciers within vegetated soils (Figure S3A,B). In barren soils, these indices were higher in PL than in GLY, but could not be statistically tested due to insufficient number of samples. In comparison, neither the richness nor Pielou's evenness of fungi demonstrated any significant differences between barren and vegetated soils (Figure 1B, Mann–Whitney *U* test, *p* = 0.22 and 0.96, respectively), nor among the various glaciers of vegetated foreland (Figure S3C,D). In barren soils, GLY samples had higher alpha‐diversity indices than PL samples, but could not be statistically tested.

Bacterial diversity indices (richness and Pielou evenness) exhibited distinct correlation patterns in barren and vegetated soils with environmental, geospatial, and climate factors (Figure 1C). In barren soils, they are significantly correlated with longitude, latitude, temperature, precipitation, and pH. In addition, richness and evenness also significantly correlated with NO_3_‐N and soil moisture, respectively. In vegetated soils, the bacterial Pielou evenness (but not richness) is significantly correlated with soil moisture and TOC. For fungi, only the evenness correlated with NO_3_‐N significantly in barren soils (Figure 1C). In vegetated soils, in contrast, only the fungal richness significantly correlated with NH_4_‐N and NO_3_‐N, but evenness did not exhibit any correlations with the measured environmental parameters.

### Taxonomic composition in glacier forelands microbial

The bacterial communities in both barren and vegetated soils were dominated by Gammaproteobacteria, Cyanobacteria, Alphaproteobacteria, Actinobacteriota, Acidobacteriota, Bacteroidota, Chloroflexi, Gemmatimonadota, Firmicutes, and Verrucomicrobiota (Figure S4A). The principal component analysis demonstrated that the community composition significantly differed between the barren and vegetated soils (Figure S4C, permutational analysis of variance [PERMANOVA], *p* = 0.002). Gammaproteobacteria was more abundant in barren soils, while Actinobacteriota, Bacteroidota, Firmicutes, Chloroflexi, and Gemmatimonadota were more abundant in vegetated soils. Among them, the relative abundances of Actinobacteriota and Bacteroidota were significantly higher in vegetated soils than those in barren soils (Mann–Whitney *U* test, *p* = 0.03 and 0.007, respectively; Figure S4A). The fungal communities in barren and vegetated soils were dominated by Ascomycota, Zygomycota, and Basidiomycota (Figure S4B). Consistent with the pattern observed in the bacterial community, fungal composition was also significantly different between barren and vegetated soils (PERMANOVA, *p* = 0.005; Figure S4D). However, the fungal communities were less separated than the bacterial community, only Zygomycota exhibited a significantly higher relative abundance in vegetated soils than that in barren soils (Mann–Whitney *U* test, *p* = 0.003; Figure S4B).

### Microbial community structure variations in glacier forelands

**Figure 2:**
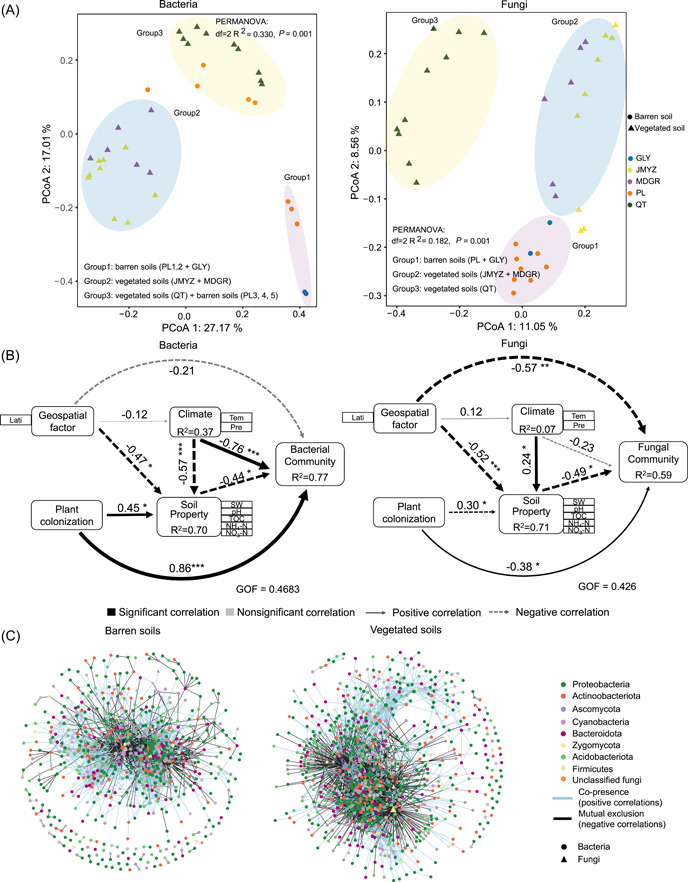
Microbial community structure variations in response to plant colonization. (A) Bacterial and fungal abundance‐weighted PCoA plots. Group1 includes barren soils from Guliya and some samples of Parlung No. 4 glaciers for bacterial community or barren soils from Guliya and PL No. 4 glaciers for fungal community; Group2 includes vegetated soils from Jiemayangzong and Mengdagangri glaciers; Group3 indicates the rest barren soils of PL No. 4 glacier and vegetated soils from Qiangtang No. 1 glacier for bacterial community or vegetated soils from Qiangtang No. 1 glacier for fungal community. (B) SEM results distinguish the effects of plant colonization, geospatial, and climate factors on bacterial and fungal community structural variations. Black lines represent significant pathways (***p < 0.001; **p < 0.01; *p < 0.05) and gray lines indicate noninsignificant pathways. Real lines represent positive effects and dashed lines indicate negative effects. The thickness of lines represents the size of the path coefficient. (C) Co‐occurrence networks of bacteria and fungi in barren and vegetated soils. Node size represents the degree, and nodes are colored by taxonomy classification. The circles represent bacterial taxa and the triangles represent fungal taxa. GLY, Guliya; GOF, goodness of fit; JMYZ, Jiemayangzong; Lati, latitude; MDGR, Mengdagangri; NO_3_‐N, soil nitrate; NH_4_‐N, soil ammonium; PCoA, principal coordinates analysis; PERMANOVA, permutational analysis of variance; pH, soil pH; PL, Parlung; Pre, precipitation; QT, Qiangtang; SEM, structural equation modeling; SW, soil moisture; Tem, temperature; TOC, soil total organic carbon.

The abundance‐weighted (Bray–Curtis distance‐based) principal coordinates analysis (PCoA) demonstrated that the bacterial communities were divided into three groups (Figure 2A and S5A; PERMANOVA, both *p* = 0.001). Group1 contains barren soils from GLY and some samples of Parlung (PL) No. 4 glaciers; Group2 contains vegetated soils from Jiemayangzong (JMYZ) and Mengdagangri (MDGR) glaciers; and Group3 contains the rest barren soils of PL No. 4 glacier and vegetated soils from Qiangtang (QT) glacier. The abundance‐unweighted (Sorensen distance‐based) bacterial community structure was used to examine community differences in OTU composition. It exhibited a similar pattern as the abundance‐weighted community structure, with three groups identified (PERMANOVA, *p* = 0.001; Figure S5B). Moreover, both abundance‐weighted and abundance‐unweighted PCoA plots demonstrated that the bacterial community was primarily separated by plant colonization on the *x*‐axis, then by geographical distance, which further separated the vegetated soils on the *y*‐axis. Additionally, the bacterial communities in the two vegetated soil groups were more similar (average similarity of 31.91%) than those between vegetated and barren soils (average similarity of 19.22% and 13.06%, respectively) (Figure S5C, Kruskal–Wallis test, both *p* < 0.001).

The fungal community exhibited a similar clustering pattern as the bacterial community, with three groups identified by both abundance‐weighted and abundance‐unweighted community metrics (Figures 2A and S6A,B, PERMANOVA, both *p* = 0.001). However, unlike the bacterial community, all barren samples of PL No. 4 glacier clustered into one group. In addition, both abundance‐weighted and abundance‐unweighted PCoA plots demonstrated that the fungal community was separated by geographical distance on the *x*‐axis, and then by plant colonization on the *y*‐axis. The community similarity among different clusters exhibited a similar pattern as the bacterial community, where the two vegetated soil groups were more similar (average similarity of 6.28%) than those between vegetated and barren soils (average similarity of 3.58% and 4.20%; Figure S6C, Kruskal–Wallis test, both *p* < 0.001). Bacterial and fungal community changes were significantly correlated (Mantel test *p* = 0.008 and <0.001 in barren and vegetated soils, respectively), indicating similar changing patterns. However, the correlation was stronger in vegetated soils (*r* = 0.43 and 0.87 for barren and vegetated soils, respectively). This is consistent with the results based on a linear correlation between PCoA axes. Specifically, significant correlations were observed between the PCoA 1 axis of bacterial and fungal communities both before and after plant colonization. However, for PCoA 2 axis, a significant correlation was only observed after plant colonization, but not before (Figure S7).

Community structure changes were further partitioned into the turnover and nestedness components. The results showed that both bacterial and fungal community structures were predominately driven by turnover (86.8% and 93.3%, respectively; Figure S8A,B). The nestedness component contributed 13.2% and 6.7% of the community structures in bacteria and fungi, respectively. In addition, the nestedness component consistently exhibited a greater contribution in barren soils than that in vegetated soils for both bacterial and fungal communities.

### Environmental drivers of bacterial and fungal communities

Variance partitioning analysis (VPA) showed that the measured environmental factors explained a slightly higher proportion of bacterial community variation (38.6%, individually explained 17.5%) than geospatial distance (26.8%, individually explained 13.3%) across all samples (Figure S9A), while climate explained 22.3% (individually explained 7.8%). For bacterial communities in barren soils, the explanation power of environmental factors (35.6%, individually explained 16.8%) was similar compared with geospatial distance (32.7%, individually explained 13.9%; Figure S9B). In comparison, after plant colonization, the contribution of environmental factors to bacterial community variations was substantially higher (53.1%, individually explained 22.4%; Figure S9C) than that in barren soils, while that of geospatial distance (34.8%) was similar to that in barren soils, but the individual explanation was substantially lower (5.6%). The contribution of climate factors to bacterial communities in vegetated soils was higher (39.8%) than that in barren soils, but the individual explanation was lower (6.3%).

For fungal community, the explanatory power of environmental factors was similar to that of geospatial distance across all samples (environmental factors explained 27.5%, individually explained 16.8%; geospatial distance explained 26.8%, individually explained 18.7%), while the climate factors explained 14.9% (individually explained 7.3%; Figure S9D). In barren soils, the fungal community was more explained by climate (20.3%, individually explained 11.3%) and geospatial distance (18.8%, individually explained 9.8%), while only 10.7% was explained by environmental factors (individually explained 9.9%), which is distinctively different from that in the bacterial community (Figure S9E). However, after plant colonization, the contributions of environmental and geospatial distance showed similar patterns to those of the bacterial community (Figure S9F). Specifically, environmental factors explained 33.4% (individually explained 21.4%) of fungal community structure, while geospatial distance only explained 17.7% (individually explained 9.6%).

Distance‐based multivariate multiple regression (DistLM) results further revealed that pH, TOC, NO_3_‐N, moisture, and NH_4_‐N were the environmental determinants of bacterial community across all samples (Table S1). In barren soils, only TOC and soil moisture significantly explained the community structure variation in bacteria. In comparison, the bacterial community in vegetated soils was explained by a collection of pH, NO_3_‐N, TOC, soil moisture, and NH_4_‐N (Table S1). The fungal community exhibited a similar pattern as the bacterial community across all samples (Table S1). However, only soil moisture significantly explained community structure in barren soils, while NH_4_‐N, NO_3_‐N, TOC, and pH significantly explained the fungal community structure in vegetated soils (Table S1).

We also performed structural equation modeling (SEM) analysis to disentangle the influence of plant colonization, geospatial, and climate factors. The bacterial community structure was explained by plant colonization, geospatial and climate factors, and plant colonization had the highest explanatory power (total path coefficient = 1.1; Figure 2B). For fungi, plant colonization and climate factors both contributed to the community structure (total path coefficients are 0.53 and 0.12, respectively), but geospatial factors had the largest explanatory power (total path coefficient is 0.82).

### Microbiome co‐occurrence network

The co‐occurrence network of bacteria and fungi was constructed for both barren and vegetated soils. The former was comprised of 670 nodes and 2748 edges, while the latter was comprised of 815 nodes and 3268 edges (Figure 2C and Table S2). Random networks were generated for both barren and vegetated samples (Table S2), and the network topology indices were significantly different from those in the observed networks. The barren soil microbiome network exhibited a similar edge per node (4.1 edges per node) compared with that in vegetated soils (4.0 edges per node), but the proportion of negative associations increased from 31.3% in barren soils to 47.7% in vegetated soils networks.

The disturbance resistance was assessed by removing nodes from the microbial networks under four scenarios (Figure S10). The microbial network of vegetated soils consistently maintained higher connectivity than that of barren soils, when the nodes were removed by decreasing betweenness, degree, and by cascading attack. This indicates that the network in vegetated soils has higher robustness (lower connectivity reduction) compared with that in barren soils. However, when the nodes were removed randomly, the patterns of connectivity reduction were similar in barren and vegetated soils.

## DISCUSSION

### Distinct influences of plant colonization on bacteria and fungi

The vegetated glacier foreland soils exhibited significantly higher carbon and nitrogen contents but lower pH than barren soils. Increased TOC with plant colonization has been consistently reported at Mendenhall Glacier, Alaska [27]; Damma glacier, central Alps [33]; and Lyman Glacier, North Cascade Mountain [23]. Significant differences in moisture, pH, and NO_3_‐N were detected among the vegetated soils. The differences can be attributed to the different vegetation types, which is consistent with the findings of Eslaminejad et al. [34] that plant species differences can cause differences in soil physicochemical properties.

Plant colonization and the associated environmental changes significantly increased bacterial alpha‐diversity (richness and evenness), but not fungal diversity (Figure 1B). Different adaptation strategies partly explain their distinct responses to plant colonization and the subsequent soil physicochemical properties change. Barren and vegetated soils shared more common bacterial OTUs (5733, 40% of the total bacterial species pool) than fungal OTUs (430, 9% of the total fungal species pool; Figure S2A). This suggests that bacteria can adapt to a wide range of environmental conditions [35, 36] and inhabit both barren and vegetated soils. These inherited bacteria from barren soils combined with bacteria that are specific to vegetated soils (46% of the total bacterial species pool) jointly lead to the increased bacterial diversity observed after plant colonization. In comparison, fungal community composition substantially shifted after plant colonization, with little shared OTUs between barren and vegetated soils (Figure S2B). This could be explained by the more specific environmental niche preference of fungi compared with bacteria [23, 37]. This result is consistent with previous findings that bacterial and plant diversity changes were significantly correlated [38], while fungal diversity remained stable [23, 39, 40, 41, 42].

The community structure of bacteria and fungi exhibited similar clustering patterns and three groups was consistently identified (Figure 2A). Barren soil samples (from GLY and PL No. 4 glaciers) clustered for both bacterial and fungal communities, despite them being distantly located in the northwest and southeast of the Tibetan Plateau, respectively (Figure 1A). This could be due to similar environmental factors (except for TOC, Table 1), which select similar microorganisms [43, 44]. Nevertheless, the bacterial community of PL glacier (barren soils) separated into two clusters while fungal was only in one cluster. This could be due to the bacterial community being more sensitive to environmental changes (Figure S9). The microbial community structures in vegetated soils were separated into two clusters for both bacteria and fungi. These clustering patterns may be attributed to the different vegetation types [45, 46, 47]. JMYZ and MDGR glaciers are both located in southern Tibet with the main vegetation being Cyperaceae, Poaceae, and Asteraceae. In comparison, the QT glacier is in central Tibet, with the main vegetation being Chenopodiaceae [1, 45, 46].

Community variations can be partitioned into nestedness and turnover components, which refers to some individuals being lost from one site to the other and the individuals of some species in one site being substituted by the same number of individuals of different species in another site, respectively [48, 49]. The bacterial community structure variation was more strongly influenced by the nestedness than the fungal community in barren soils (Figure S8), indicating higher shared bacterial phylotypes among samples, which is explained by the higher dispersal capacities of bacteria [20, 50]. This also explains the high similarity of the bacterial community among the barren soil samples despite the two glacier forelands being nearly 1600 km apart (Figure 1A). In comparison, fungi are more susceptible to dispersal limitations due to their larger cellular size [5, 20, 50], thus fungi are more endemic, which led to a higher contribution of turnover. In comparison, the relative contributions of nestedness and turnover were similar between bacterial and fungal communities in vegetated soils (Figure S8). This could be explained by the enhanced environmental selection post plant colonization, which selects for specific microorganisms that are adapted to the soils with higher organic carbon and lower pH due to plant colonization [23].

### Plant colonization influences the community assembly processes of bacterial and fungal communities

pH is one of the most important environmental drivers of bacterial diversity in soils, determining the balance between stochastic and deterministic processes in glacier forelands [24, 51, 52, 53, 54]. In the present study, pH influenced bacterial community structure across all samples and in vegetated soils, but not in barren soils (Table S1). This is consistent with our hypothesis one, thus indicating that the influence of pH could be associated with plant colonization. Bacterial community structure in barren soils was mainly explained by TOC and soil moisture. This is consistent with the glacier foreland soils being carbon‐limited [15, 25, 26], whereas soil moisture is an indicator of soil development [55]. In vegetated soils, pH exhibited the highest explaining power among the measured environmental factors. Plant colonization lowers pH due to litter input and root exudates [23, 29, 33]. Thus, the results of Tripathi et al. [24] could predominately occur post plant colonization, or during the transition before and after plant colonization, thus the role of plant colonization in glacier foreland could be overlooked.

Although fungal community variation was less explained by environmental factors (Table S1 and Figure S9), pH also significantly explained fungal community across all samples and in vegetated soils but with much lower explanatory power compared with that for bacterial community (Table S1). This indicates that pH also mediates fungal community changes, not only bacterial community as reported previously [56, 57]. In addition, this also suggests pH played a weaker role in fungal community than that in bacterial community, which could be due to fungi generally exhibit wider pH ranges for growth [58].

Plant colonization also enhanced the community structure covariation between bacterial and fungal communities, which could be explained by the strong environmental filtering effects of plants [23, 33]. In barren soils, the environmental filtering on the fungal community was particularly weak, with only 15.3% (fungal) of the community variations being explained by the measured physicochemical factors (Table S1). In comparison, plant colonization enhanced soil carbon and nitrogen concentration and lowered pH (Table 1). These soil physicochemical properties changes act as a strong environmental selection pressure [59, 60], and only allow the survival of microbiomes that can adapt to these environmental conditions.

We disentangled the influence of plant colonization, geospatial and climate factors using SEM. Plant colonization (but not geospatial and climate factors) was the dominated driver of bacterial community (Figure 2B). Geospatial factors only had an indirect effect and the effect was weaker than that of plant colonization. The lack of direct influence can be explained by the high dispersal capacity of bacteria, which makes them less affected by geospatial separation [20, 50], while the indirect influence can be explained by spatial heterogeneity. In addition, climate factors (temperature and precipitation) also influenced bacterial community structure via both direct and indirect effects, but the effect was still weaker than plant colonization. This is consistent with the results that temperature and precipitation can change the structure of the bacterial community by affecting the soil physicochemical properties [61, 62, 63]. In contrast, fungal community was influenced by both the direct and indirect effects of geospatial factors and plant colonization, but the effect of plant colonization on fungal community was weaker than that of geospatial factors, which was also lower than that of bacterial community. This can be explained by the stronger dispersal limitations of fungi than that of bacterial due to their larger cellular size [5, 20, 50]. The climate factors influence fungal communities only via indirect effects, and the effect was still weaker than plant colonization and that of bacterial. This result is consistent with Yang and Wu which demonstrated that the fungal community is more stable in response to climate factors changes than that of bacterial community [64].

### Plant colonization stabilizes microbial communities by enhancing competition in glacier foreland

Plant colonization altered the topology of the microbial co‐occurrence network (Table S2). Microbial interactions in barren soil networks were dominated by positive correlations (Figure 2C). Positive interactions represent two species occupying similar ecological niches and response to external disturbances in a similar pattern or two microbes form syntrophic relations [65]. Moreover, microbial interactions are resource availability‐dependent [66], and oligotrophic environments can enhance collaborative substrate degradation, such as cellulose or humic acids. Thus, the lower nutrients in barren soils before plant colonization may promote positive or copresence relationships among bacteria and fungi. In comparison, plant colonization increased the number of correlations within the network and increased the proportion of negative associations (Figure 2C). Negative associations usually represent strong competitive relationships over nutrients or ecological niches between microorganisms [65]. Our results revealed increased interkingdom competitions after plant colonization (from 4.5% to 18% of total correlations). This may suggest a shift from reciprocal symbiosis to competitive relationships for a shared resource after plant colonization.

The network stability of the microbial community was enhanced by plant colonization (Figure S10). The lower stability in the positive association‐dominated barren soil network is consistent with a previous study in Antarctic snow microbiomes [67]. Positive association indicates microorganisms occupy similar ecological niches and respond to environmental stimuli similarly. Thus, the disruption of one microorganism can quickly spread and destabilize the entire network [68]. By contrast, a network composed of both positive and negative correlations can mitigate the effects caused by such disturbances and enhance the stability of the network [69]. Therefore, plant colonization enhances the complexity and stability of the microbial network, which makes the network more robust and enhances the resistance to environmental disturbances. Furthermore, there were fewer leaps in the node removal robustness tests (Figure S11). This suggests that the network in the vegetated soils could have less critical microorganisms for the network integrity and thus can be more robust when “targeted” attack on core microorganisms, further supporting the microbial communities become more resistant to extreme disturbances [70].

### Technical limitations and future suggestions

The present work relies on the short amplicon sequencing targeting the V4–V5 hypervariable regions of the 16S ribosomal RNA (rRNA) gene. There is inherited limitation in profiling microbial community using short amplicon reads such as limited taxonomic resolution and over‐ or underestimation of microbial diversity [71]. High‐throughput sequencing targeting the entire rRNA operon can provide potentially strain‐specific identification, revealing the hidden microbial diversity [72, 73]. This could be particularly valuable for glacier foreland ecosystems where a large number of sequences are poorly resolved at lower taxonomic resolution. Thus, further studies using the third generation sequencing platforms such as PacBio and nanopore may improve community dynamic profiling accuracy in glacier foreland.

## CONCLUSIONS

Here, we examined the bacterial and fungal community biogeography in glacier foreland soils across the Tibetan Plateau. In Tibetan glacier forelands, plant colonization is a strong environmental filtering factor for the biogeography of the microbial community in glacier foreland, suggesting the role of pH and SOC in microbial community dynamics is the result of plant colonization. Plant colonization alters diversity, community structure, response to environmental factors, and biotic interactions in bacterial and fungal communities. The present study broadens the understanding of microbial community dynamics at glacier foreland, and emphasized the vital roles of plant colonization on nutrient accumulation and ecosystem stability from a microbial point of view.

## MATERIALS AND METHODS

### Sample collection

Soil samples were collected between 2018 and 2020 in five geographically separated glacier forelands located in JMYZ (southern Tibet, 82°12′ E, 30°15′ N), MDGR (southern Tibet, 90°35′ E, 28°28′ N), QT No. 1 (central Tibet, 88°25′ E, 33°10′ N), PL No. 4 (southeast of Tibet, 96°156′ E, 29°15′ N), and GLY glaciers (north‐west of Tibet, 81°30′ E, 35°12′ N), respectively (Figure 1A and S11). Soil samples were collected as close to the glacier terminus as possible, as it was impossible to reach the terminus of some glaciers due to logistic issues. The soils collected in PL (0.4–1.9 km from the glacier terminus, sites = 5, *n* = 8) and GLY (0.5 km from the glacier terminus, site = 1, *n* = 2) had no plant colonization, and thus were grouped as barren soils. The soils collected in MDGR (1.2–2.6 km from the glacier terminus, sites = 3, *n* = 6), QT (0.8–1.6 km from the glacier terminus, sites = 3, *n* = 10), and JMYZ (4.8–5.8 km from the glacier terminus, sites = 3, *n* = 9) had vegetation, thus were grouped as vegetated soils. During vegetated soil collection, we collected bulk soils and avoided collecting soils near plants to avoid the rhizosphere effect. Due to the low microbial content of soil samples at some sites, the number of repeats was reduced. All sampling sites were located at least 10 m away from glacier runoff to avoid the influence of glacial meltwater. A 50 × 50 cm square was randomly selected at each sampling site, three points on the diagonal were picked to collect surface soil samples (0–5 cm depth) and mixed. A total of 100 g soil was collected at each sampling site. The soils were sieved through an ethanol‐sterilized 2‐mm mesh to remove stones and plant materials (if any) in the field, and then divided into two equal portions for DNA extraction and physicochemical analyses. Soil samples were transported to the laboratory in an insulated box at 4°C. Soils for DNA extraction were then stored at −80°C until used, and those for physicochemical analyses were air‐dried.

### Soil physicochemical analysis

Briefly, soil physicochemical properties were analyzed using standard methods [74]. Soil moisture was determined by gravimetrical methods after soils were oven‐dried at 105°C for 72 h [75]. Soil pH and conductivity were measured in a 1:2.5 soil‐to‐water suspension using a pH meter (PHS‐3C) [76]. TOC was measured in the solid state using a TOC analyzer (TOCVCPH) [76], while the concentrations of nitrate nitrogen (NO_3_‐N) and ammonium nitrogen (NH_4_‐N) were measured using an elemental analyzer (Smartchem200, AMS) [77].

### DNA extraction, polymerase chain reaction (PCR), and high‐throughput sequencing

Total DNA was extracted from 0.5 g soil using the MO BIO Power Soil DNA extraction kit (Mo Bio Laboratories) according to the manufacturer's instructions. The concentration of the extracted DNA was estimated using a Qubit (Qubit 4.0, Thermo Fisher Scientific). Universal primers 515F (5′‐GTGCCAGCMGCCGCGGTAA‐3′) and 907R (5′‐CCGTCAATTCMTTTRAGTTT‐3′) were used to amplify the V4–V5 hypervariable regions of the 16S rRNA gene [78]. For fungi, the primers pair ITS1‐1F‐F (5′‐CTTGGTCATTTAGAGGAAGTAA‐3′) and ITS1‐1F‐R (5′‐GCTGCGTTCTTCATCGATGC‐3′) were used to amplify the ITS1 region [79]. The PCR mixture (25 μL) contained 1 × PCR buffer, 1.5 mM of MgCl_2_, 0.4 μM each of deoxynucleoside triphosphate base, 1.0 μM of each primer, 0.5 U of Ex Taq (Takara), and 20 ng of DNA template. The PCR program for the 16S rRNA gene was the following: 95°C for 3 min followed by 27 cycles of 95°C for 30 s, 55°C for 30 s, and 72°C for 45 s, with a final extension at 72°C for 10 min. For the ITS1 region, the PCR program was the follow: 98°C for 1 min followed by 30 cycles of 98°C for 10 s, 50°C for 30 s, and 72°C for 30 s, with a final extension step of 5 min at 72°C. To minimise PCR batch‐to‐batch variations and maximize PCR product quantity, triplicate PCR reactions were conducted for the same sample and the PCR products were then pooled for purification using the OMEGA Gel Extraction Kit (Omega Bio‐Tek) following electrophoresis. PCR products from different samples with unique barcodes were pooled in equal molar amounts, and then used for pair‐end sequencing (2 × 250 bp) on an Illumina HiSeq sequencer (PE250) at the Magigene Biotechnology Co., Ltd.

### Data processing

The sequencing reads were processed using the USEARCH pipeline (v. 10.0.259) [80]. Paired‐end reads were merged, and sequences were quality‐screened with the following settings: sequences with either 2 bp mismatches with the primer, 1 bp mismatch with the barcode, homopolymers longer than 8 bp or a maximum expected error probability >0.5 were removed from the analysis. To improve fungal identification, the fungal ITS1 sequence was extracted using the ITSx algorithm [81]. Thereafter, all sequences were classified into OTUs at 97% identity. The sequences were classified using Wang's method against the Silva database (2019.12, release 138), with a minimum confidence score of 80% [82]. Then all eukaryota, chloroplasts, mitochondria, and unknown sequences were removed. The samples were randomly subsampled without replacement to an equal depth of 14,473 for bacteria and 4789 for fungi (the smallest sample size across the entire data set). This was to ensure samples had the same sequencing depth, which makes diversity indices comparison at a consistent scale.

### Statistical analysis

The soil physicochemical properties between barren and vegetated soils were compared using the Mann–Whitney *U* test in the rstatix R package [83], the differences of those among glaciers in vegetated soils were compared using Kruskal–Wallis test in the stats R package. In addition, differences among glaciers within barren soils were not compared due to the lower sample size of GLY. The richness (number of OTUs) and evenness indices were calculated from the OTU table using the “diversity” function in the vegan R package [84]. The alpha‐diversity indices (richness and evenness) in barren and vegetated soils were compared using the Mann–Whitney *U* test in the rstatix R package [83]. The correlation analysis between alpha‐diversity indices and environmental factors was performed using “cor” function (Spearman correlation) in the stats R package [85] and the results were visualized using a correlation heatmap in the corrplot R package [86]. The community structure differences between samples were calculated using both the abundance‐weighted (Hellinger‐transformed Bray–Curtis dissimilarity) and abundance‐unweighted (Sorensen distance) matrices, and the results were visualized using PCoA plots. The community structure changes were further partitioned into the turnover and nestedness components using “beta. pair” function in the betapart R package [87]. To distinguish the effects of plant colonization, geospatial (latitude), climate (temperature and precipitation), and soil physicochemical factors (moisture, pH, TOC, NH_4_‐N, and NO_3_‐N), SEM was carried out using the “plspm” function from the plspm R package [88] and the data used in the model was homogenized. In SEM, the PCoA scores of the first axis were used to represent bacterial and fungal community structures. In addition, the path coefficient of SEM represents the degree to which the predictor contributes to the response variable, the positive coefficient indicates a positive effect, while negative coefficient indicates a negative effect. In addition, the goodness of fit value is used to measure the fit of the model and the model fits better when the value is larger. We construct the SEM based on the following assumptions. (1) Plant colonization changes soil physicochemical properties, such as soil pH, moisture, and nutrient content [23, 29, 89]. (2) Both plant colonization and soil physicochemical properties affect the microbial community structure [23, 24, 25]. (3) The spatial distribution of glaciers can also affect climate and microbial communities [90, 91]. (4) The climate factors (temperature and precipitation) can influence soil physicochemical properties and microbial community [92, 93]. The temperature and precipitation data used in the SEM were derived from the National Tibetan Plateau Data Center [94, 95]. The VPA was used to partition the community variation with environmental, geospatial, and climate factors, DistLM analysis was used to further analyze the associations between microbial community structure and the measured soil physicochemical factors. PERMANOVA was used to test the significance of community structure differences of the three clusters identified in the PCoA plot and also the influence of plant colonization [96] using “adonis2” function in the vegan R package [84].

### Network analysis

To identify the impact of plant colonization on microbial interactions, CoNet [97] was used to construct co‐occurrence networks for the bacterial and fungal communities. To reduce the false correlation discovery rate and reduced network complexity, only OTUs that occurred in more than 50% of samples were selected for network construction [98]. Briefly, the distribution of all pairwise scores was computed for the five recommended similarity measures (Kullback–Leibler and Bray–Curtis dissimilarities, Pearson and Spearman correlations, and mutual information) to ensure the consistency of correlations. The top 1000 positive and 1000 negative edges supported by all five measures were retained initially. Then, 500 random permutations (with renormalization for correlation measures) and bootstrap scores were generated for each measurement and edge, following the ReBoot routine. The measure‐specific *p* value was then computed and then merged using Brown's method. After applying Benjamini–Hochberg's false discovery rate correction, edges with merged *p* < 0.05 were kept. Any edge for which the five measures did not agree on the interaction type (i.e., positive or negative, respectively) or whose initial interaction type contradicted the interaction type determined with the *p* value was also discarded. Edges with scores outside the 95% confidence interval defined by the bootstrap distribution or not supported by all five measures were also discarded. To avoid the effect of different samples of microbial co‐occurrence networks in barren and vegetated soils, we randomly selected the same number of samples (10 samples) in vegetated soils and constructed three random networks using the same methods described above. They exhibited similar characteristic features with the vegetated network but substantially differed from the barren network (Table S2). Thus, only the results of vegetated network using all samples were reported in the manuscript, vegetated networks with the same sample numbers are provided in the Supporting Information file (Table S2). The networks were then visualized using Cytoscape (v. 3.9.0) [99] and nodes were organized using the Fruchterman–Reingold algorithm. Positive cohesion, derived from positive pairwise correlations or copresence relationships, could reflect the degree of cooperative behaviors, whereas negative cohesion could indicate the magnitude of competitive behaviors [100]. Network topologies (such as the node degree, clustering coefficient, and transitivity) were calculated in the R environment using the “igraph” package [101]. We also constructed 500 random networks for both barren and vegetated soils using “randnet” function in the NetworkToolbox R package with the same number of nodes and edges as the observed networks. Then network topology statistics was also calculated using the “igraph” package.

Network robustness is the ability of a network to maintain a certain level of structural integrity and its original functions after being attacked, which is the key to whether the damaged network can continue to operate normally [102]. The robustness of the barren and vegetated soils networks was tested using the “NetSwan” package [103] in the R environment. The network robustness was measured by the loss in network connectivity under four different node removal scenarios: random removal, removal by decreasing the order of degree, betweenness, and under the cascading scenario.

## AUTHOR CONTRIBUTIONS

Mukan Ji and Yongqin Liu designed the study; Yang Liu analysed data and wrote the manuscript with Mukan Ji; Wenqiang Wang collected samples; Tingting Xing and Qi Yan contributed to soil DNA extraction and physicochemical analysis; Belinda Ferrari substantially reviewed the manuscript; All authors discussed the results and contributed to the final manuscript.

## CONFLICT OF INTEREST STATEMENT

The authors declare that they have no conflict of interest.

## Supporting information

Supporting information.

## Data Availability

Supplementary materials (figures, tables, scripts, graphical abstract, slides, videos, Chinese translated version and update materials) may be found in the online DOI or iMeta Science http://www.imeta.science/. The sequencing raw sequences have been submitted to NCBI Short Read Archive (SRA) with accession number of PRJNA823396 (SRR18614372‐SRR18614406) and which also deposited in the Genome Sequence Archive in the National Genomics Data Center, China National Center for Bioinformation/Beijing Institute of Genomics, Chinese Academy of Sciences with accession number of CRA009658 (SAMC1059968‐SAMC1060002).
