# Supplementary material for: Plant colonization mediates the microbial community dynamics in glacier forelands of the Tibetan Plateau
Source: Imeta. 2023 Feb 14;2(1):e91. doi: 10.1002/imt2.91 (PMC10989783; doi:10.1002/imt2.91)
Supplement: Supplementary file 1 — Supporting information. [file IMT2-2-e91-s001.docx]

Supplementary material

Table S1. DistLM results of bacterial and fungal communities in barren and vegetated soils. The bold *P* value indicates that this soil physiochemical factor has a significant effect on the microbial community.

|  | **Bacteria** | | | | | |  | **Fungi** | | | | | |
| --- | --- | --- | --- | --- | --- | --- | --- | --- | --- | --- | --- | --- | --- |
|  | **All samples** | | **Barren soils** | | **Vegetated soils** | |  | **All samples** | | **Barren soils** | | **Vegetated soils** | |
|  | *P* value | R^2^ | *P* value | R^2^ | *P* value | R^2^ |  | *P* value | R^2^ | *P* value | R^2^ | *P* value | R^2^ |
| pH | **0.001** | 0.1872 | 0.317 | 0.0881 | **0.001** | 0.2190 |  | **0.001** | 0.0572 | 0.063 | 0.1455 | **0.040** | 0.0745 |
| Moisture | **0.001** | 0.0459 | **0.038** | 0.1695 | **0.002** | 0.0779 |  | **0.002** | 0.0457 | **0.034** | 0.1529 | 0.076 | 0.0794 |
| TOC | **0.001** | 0.0989 | **0.009** | 0.2774 | **0.002** | 0.0755 |  | **0.001** | 0.0433 | 0.504 | 0.0987 | **0.014** | 0.0884 |
| NH_4_-N | **0.004** | 0.0394 | 0.386 | 0.0801 | **0.004** | 0.0654 |  | **0.002** | 0.0455 | 0.433 | 0.1073 | **0.002** | 0.1153 |
| NO_3_-N | **0.014** | 0.0637 | 0.469 | 0.0746 | **0.001** | 0.1060 |  | **0.001** | 0.0622 | 0.501 | 0.0942 | **0.002** | 0.0949 |

|  |  | Barren soils | Vegetated soils | Vegetated network with 10 random samples  (Random network 1) | Vegetated network with 10 random samples  ( Random network 2) | Vegetated network with 10 random samples  ( Random network 3) | Barren soils random network | Vegetated soils random network |
| --- | --- | --- | --- | --- | --- | --- | --- | --- |
| Nodes |  | 670 | 815 | 1164 | 1706 | 1283 | - | - |
|  | Bacteria | 654 | 795 | 1124 | 1646 | 1231 | - | - |
|  | Fungi | 16 | 20 | 40 | 60 | 52 | - | - |
| Edges |  | 2748 | 3268 | 3113 | 916 | 3401 | - | - |
|  | Positive | 1887 (68.7%) | 1710 (52.3%) | 1879 (60.4%) | 3340 (56.5%) | 1935 (56.9%) | - | - |
|  | Negative | 861 (31.3%) | 1558 (47.7%) | 1234 (39.6%) | 2576 (43.5%) | 1466 (43.1%) | - | - |
| Edges per node |  | 4.1 | 4.0 | 2.7 | 3.5 | 2.7 | - | - |
| Edge distribution |  |  |  |  |  |  | - | - |
| Bacteria to bacteria | Positive | 1867 | 1704 | 1792 | 3217 | 1718 | - | - |
|  | Negative | 726 | 971 | 888 | 1792 | 1083 | - | - |
| Fungi to fungi | Positive | - | - | 6 | 6 | 14 | - | - |
|  | Negative | 11 | 12 | 19 | 66 | 17 | - | - |
| Bacteria to fungi | Positive | 20 | 6 | 81 | 117 | 203 | - | - |
|  | Negative | 124 | 575 | 327 | 718 | 366 | - | - |
| Clustering coefficient |  | 0.276 | 0.255 | 0.262 | 0.271 | 0.067 | 0.012+0.001 | 0.010+0.001 |
| Network density |  | 0.016 | 0.011 | 0.007 | 0.008 | 0.006 | 0.012+0.001 | 0.010+0.001 |
| Transitivity |  | 0.27 | 0.15 | 0.24 | 0.36 | 0.26 | 0.012+0.001 | 0.010+0.001 |

Table S2. Topological properties of the co-occurrence networks of bacteria and fungi in barren and vegetated soils and those of the three random networks in vegetated soils and random networks for barren and vegetated soils.


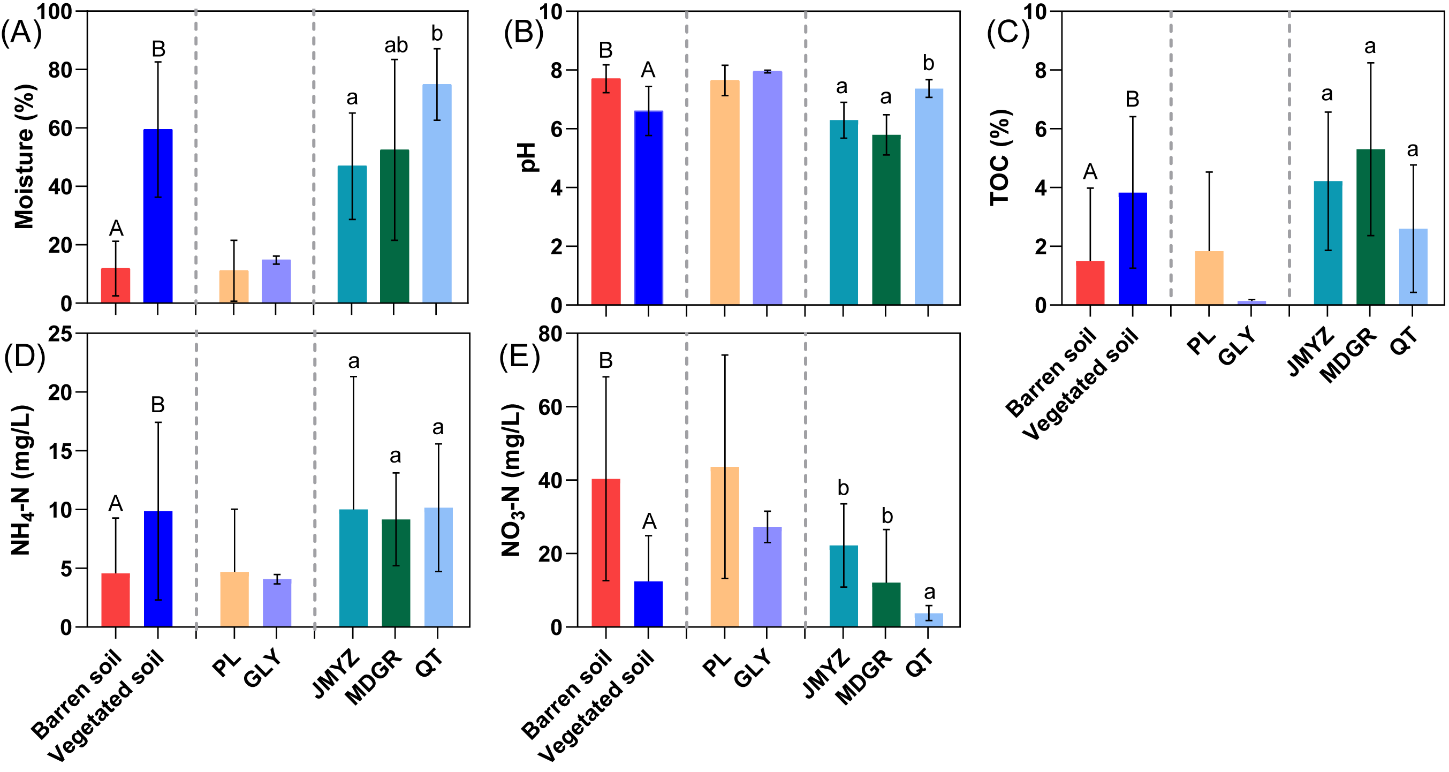


Figure S1. Soil physicochemical properties comparison between barren and vegetated soils, and among glaciers within barren or vegetated soil groups. (A) soil moisture; (B) soil pH; (C) total organic carbon; (D) ammonium nitrogen (NH_4_^+^-N); (E) nitrate nitrogen (NH_4_^-^-N) Capital letters indicate significant differences between barren and vegetated soils based on Kruskal-Wallis test and Dunnett T3 post-hoc analysis; lowercase letters indicate significant differences among glaciers within barren or vegetated soil group. PL: Parlung glacier, GLY: Guria glacier, JMYZ: Jiemayangzong glacier, MDGR: Mengdagangri glacier, QT: Qiangtang NO.1 glacier.


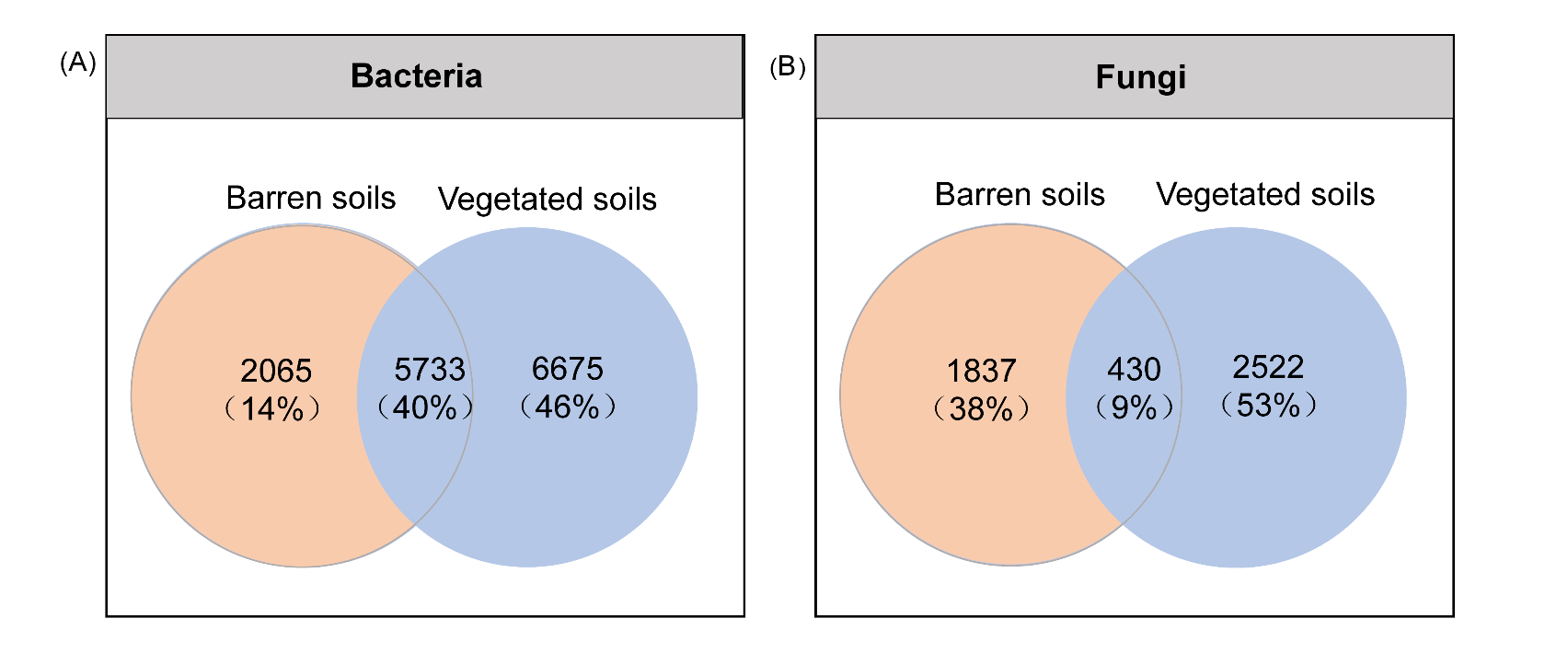


Figure S2. The variation of γ-diversity in bacterial (A) and fungal (B) community. Yellow circle represents the number of OTUs specific to barren soils, blue circle represents the number of OTUs specific to vegetated soils, and the middle part represents the number of OTUs shared by both.


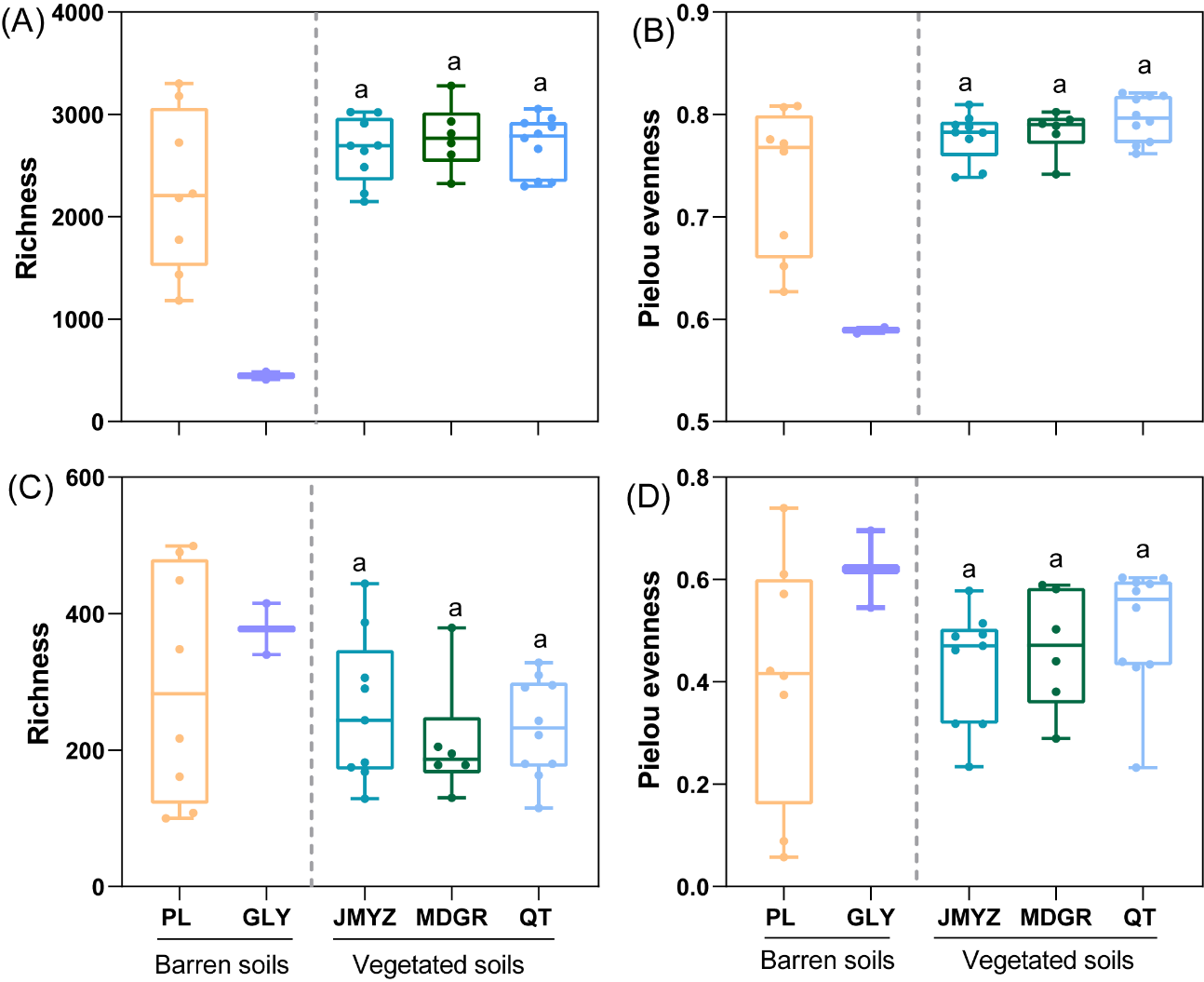


Figure S3. Variation in bacterial (A-B) and fungal (C-D) diversity indices across glaciers within barren and vegetated soil groups. Letters indicate significance at *P* < 0.05 based on Kruskal-Wallis test and Dunnett T3 post-hoc analysis. PL: Parlung glacier, GLY: Guria glacier, JMYZ: Jiemayangzong glacier, MDGR: Mengdagangri glacier, QT: Qiangtang NO.1 glacier.


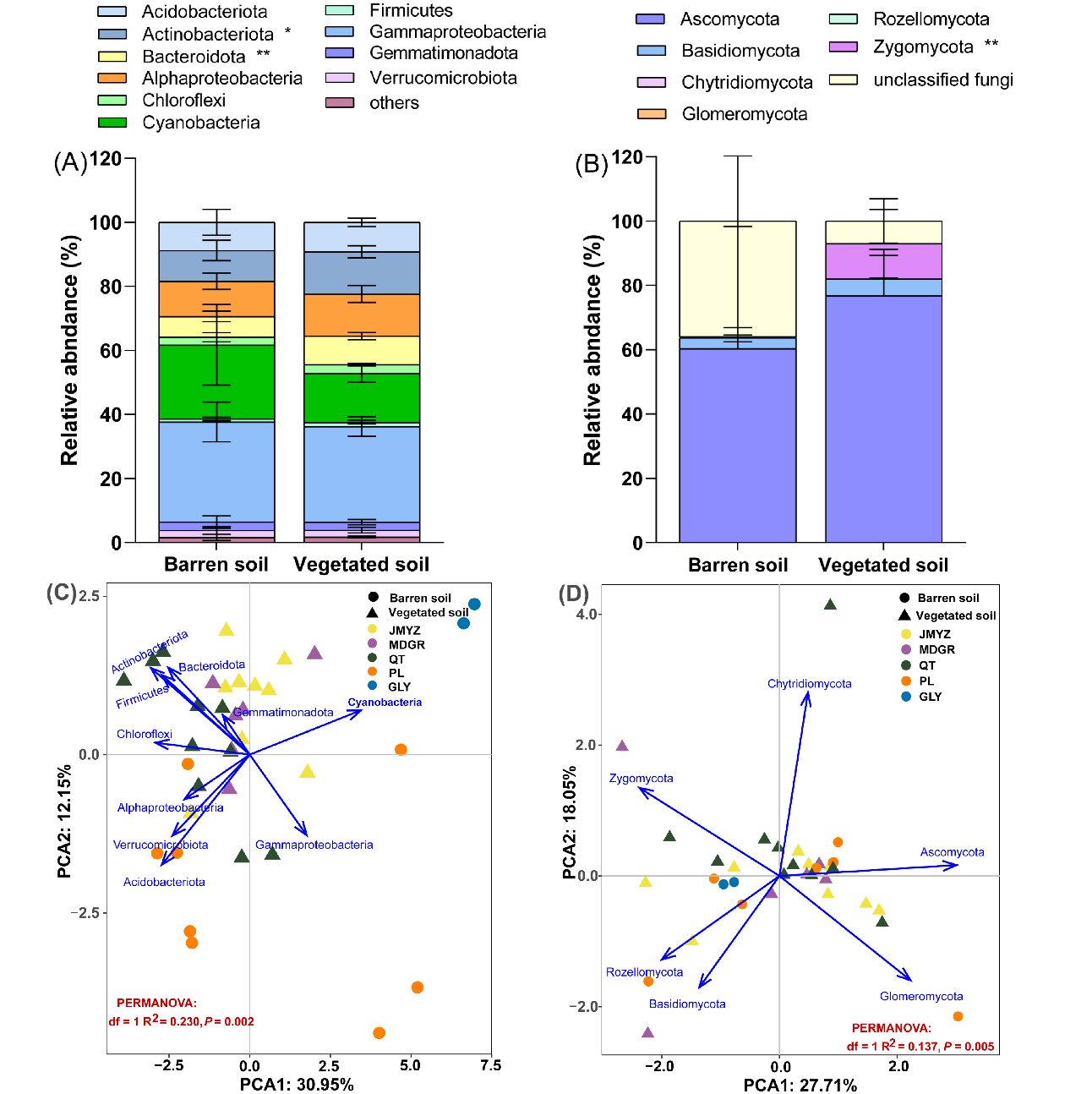


Figure S4. Community composition of bacterial and fungal communities. (A) community composition of bacteria in barren and vegetated soils at the phylum level. (B) Community composition of fungi in barren and vegetated soils at the phylum level. (C) principal coordination analysis (PCA) ordination plot of bacterial community at the phylum level. (D) PCA ordination plot of fungal community at the phylum level. Asterisk indicate significant differences between barren and vegetated sols (Mann-Whitney U test, ***, *P* < 0.001; **, *P* < 0.01; *, *P* < 0.05). JMYZ: Jiemayangzong glacier, MDGR: Mengdagangri glacier, QT: Qiangtang NO.1 glacier, PL: Parlung glacier, GLY: Guria glacier.


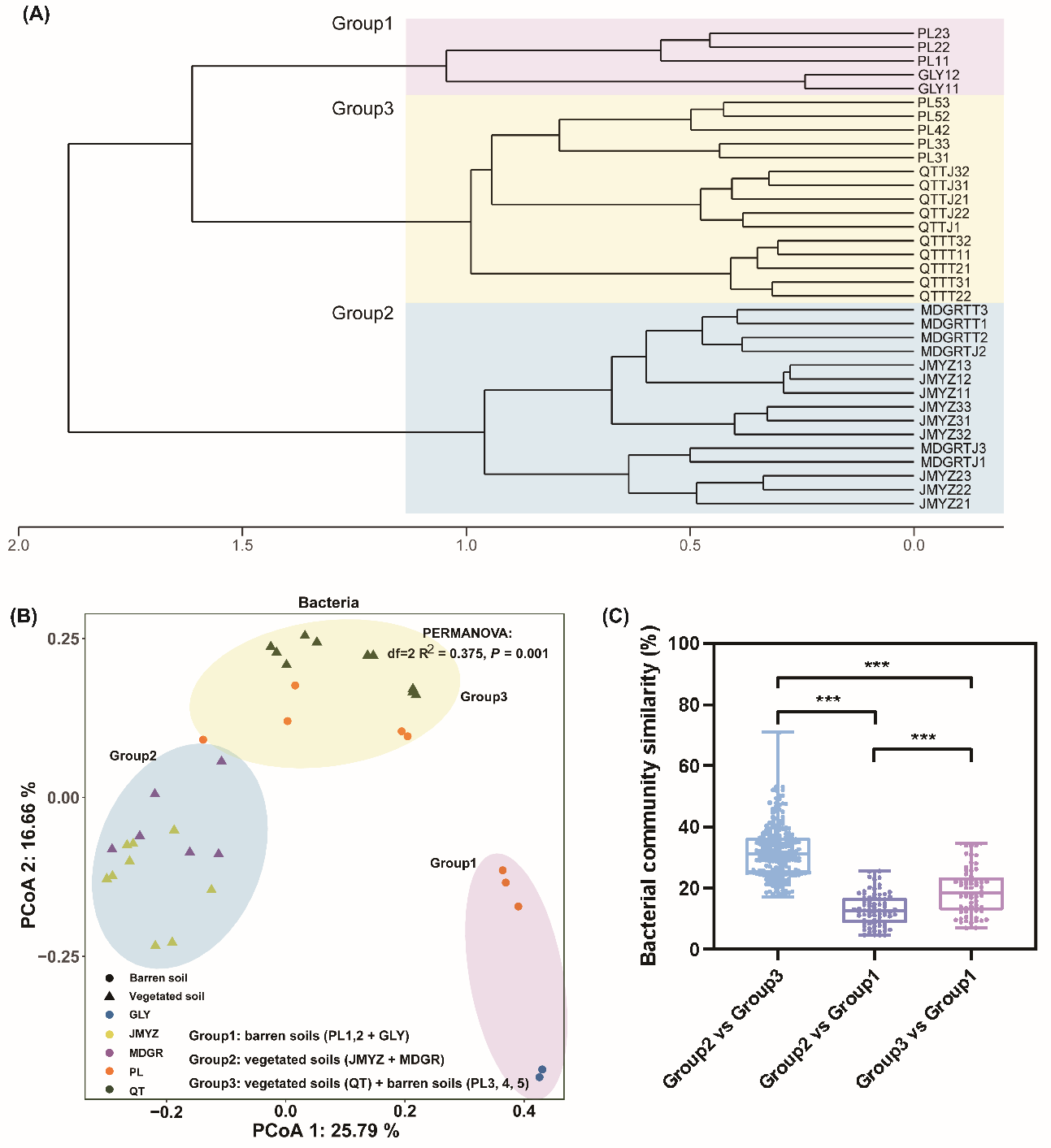


Figure S5. Bacterial community structure variations in barren and vegetated soils. (A) Cluster analysis of bacterial community; (B) Principal coordinates analysis of the abundance-unweighted (Sorensen distance-based) bacterial community; (C) Community similarity analysis among different community clustering groups. Asterisk indicate significant differences based on Permanova test (***, *P* < 0.001; **, *P* < 0.01; *, *P* < 0.05). PL: Parlung glacier, GLY: Guria glacier, JMYZ: Jiemayangzong glacier, MDGR: Mengdagangri glacier, QT: Qiangtang NO.1 glacier. Group1 includes barren soils from Guliya and some samples of Parlung NO.4 glaciers; Group2 includes vegetated soils from Jiemayangzong and Mengdagangri glaciers; Group3 indicate the rest barren soils of Parlung NO.4 glacier and vegetated soils from Qiangtang glacier.


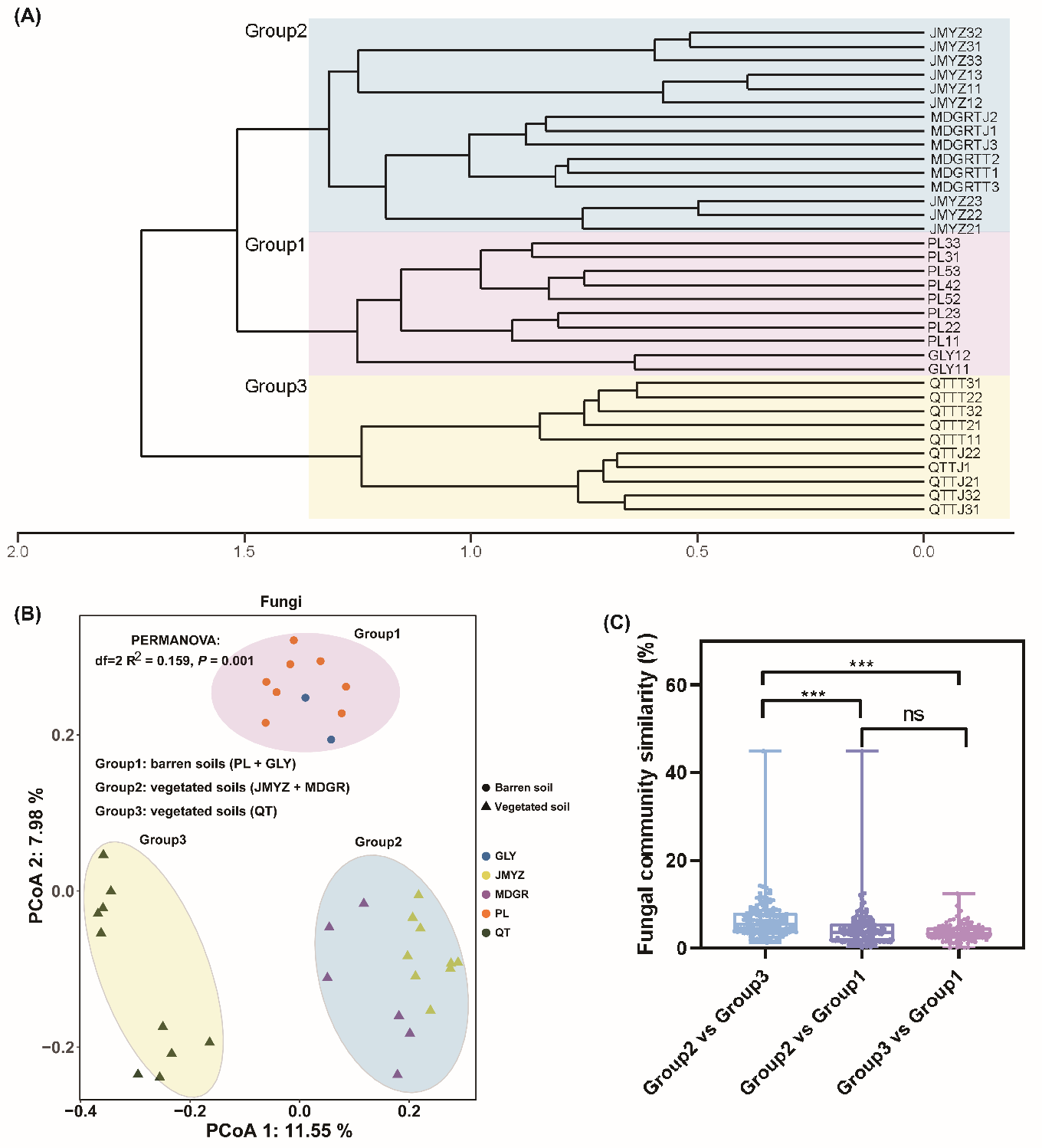


Figure S6. Fungal community structure comparison in barren and vegetated soils. (A) Cluster analysis of fungal communities; (B) Principal coordinates analysis of the abundance-unweighted (Sorensen distance-based) fungal communities; (C) Community similarity analysis among different clustering groups. Asterisk indicate significant differences based on Permanova test (***, *P* < 0.001; **, *P* < 0.01; *, *P* < 0.05). PL: Parlung glacier, GLY: Guria glacier, JMYZ: Jiemayangzong glacier, MDGR: Mengdagangri glacier, QT: Qiangtang NO.1 glacier. Group1 includes barren soils from Guliya and Parlung NO.4 glaciers; Group2 includes vegetated soils from Jiemayangzong and Mengdagangri glaciers; Group3 indicate vegetated soils from Qiangtang glacier.


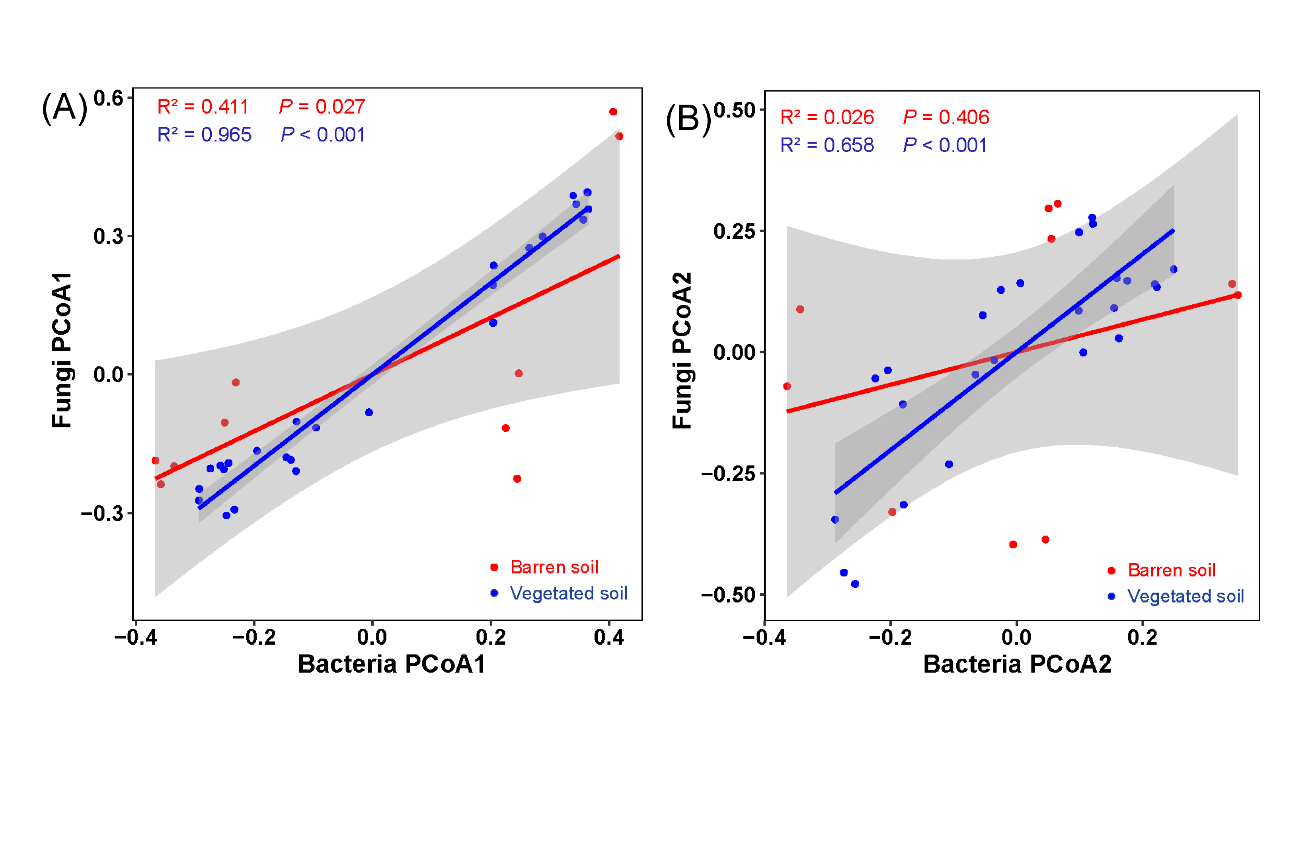


Figure S7. Pearson correlation analysis of PCoA scores of x- (A) and y- axes (B) between bacterial and fungal communities. Red and blue lines indicate the barren and vegetated soils, respectively. The gray areas around the fitted straight line indicate the 95% confidence interval.


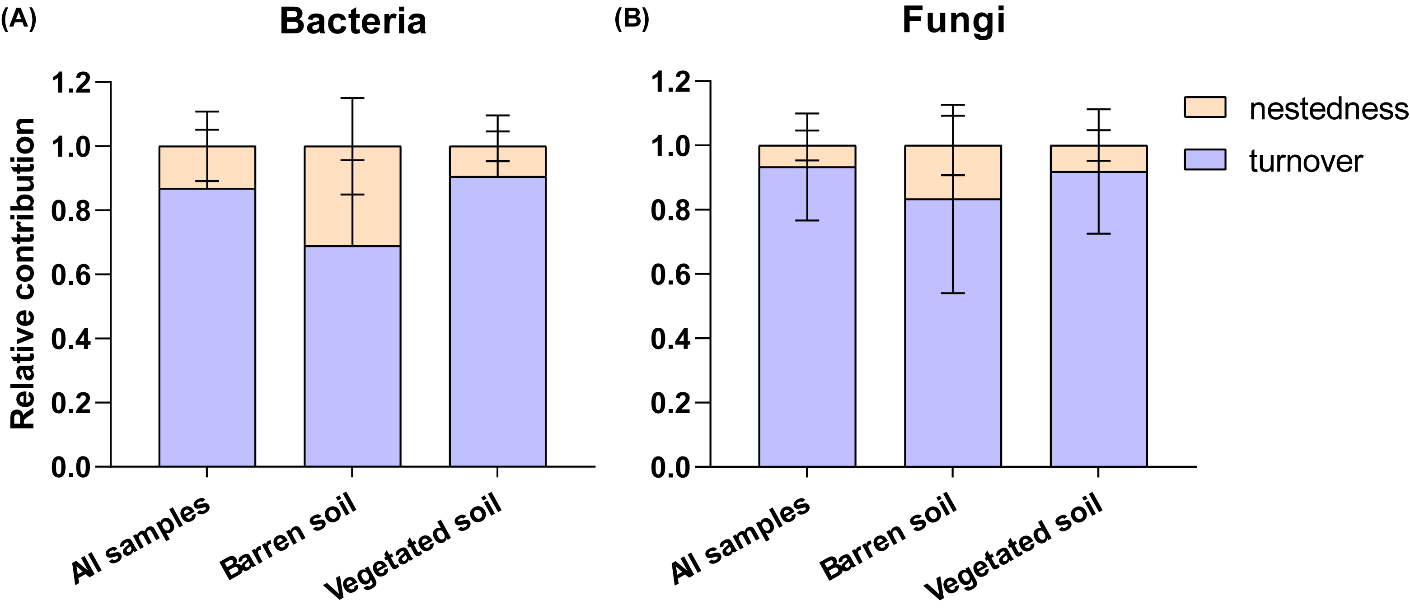


Figure S8. The relative contribution of turnover and nestedness components in bacterial (A) and fungal communities (B). All samples refer to the relative contributions of turnover and nestedness to the overall bacterial or fungal community. Barren soils refer to the relative contribution of turnover and nestedness components to the bacterial or fungal community in barren soils. Vegetated soils refer to the relative contribution of turnover and nestedness to bacterial or fungal community in the vegetated soils.


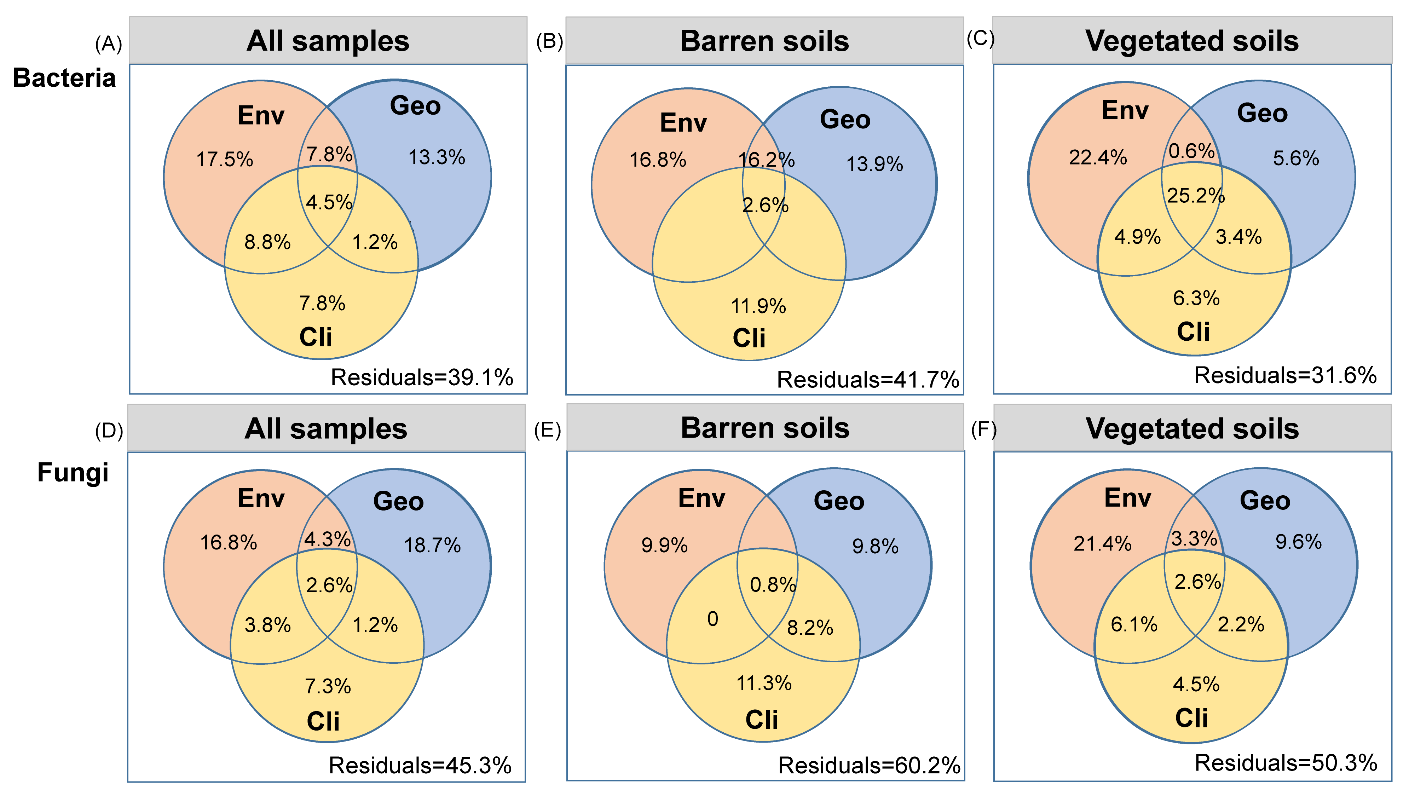


Figure S9. VPA analyses show the relative contribution of environmental and geospatial factors to bacterial (A-C) and fungal (D-F) community variations. Env, soil physicochemical properties (pH, soil moisture, TOC, NH_4_-N, and NO_3_-N); Geo, geospatial factors (longitude, and latitude); Cli, climate factors (temperature, and precipitation).


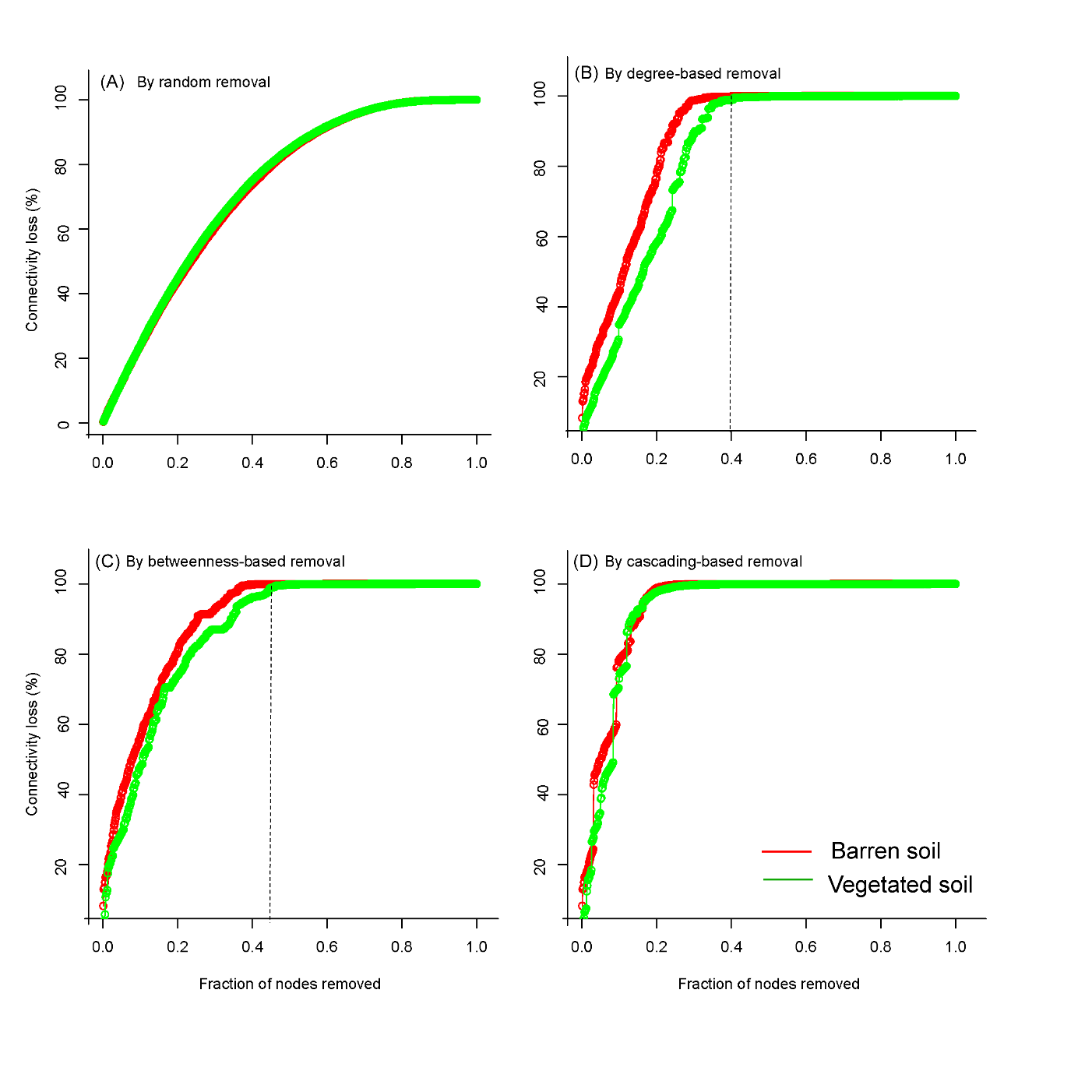
Figure S10. The robustness test of networks in barren and vegetated soils.

The network robustness refers to the capability of maintaining network connectivity under node loss. It is tested by removing nodes randomly (A), reducing order of degree (B), reducing order of betweenness (C), and cascading effect (D). Dashed line indicates the fraction of nodes removed when the connectivity reduction in vegetated soils network similar to that in barren soils network.


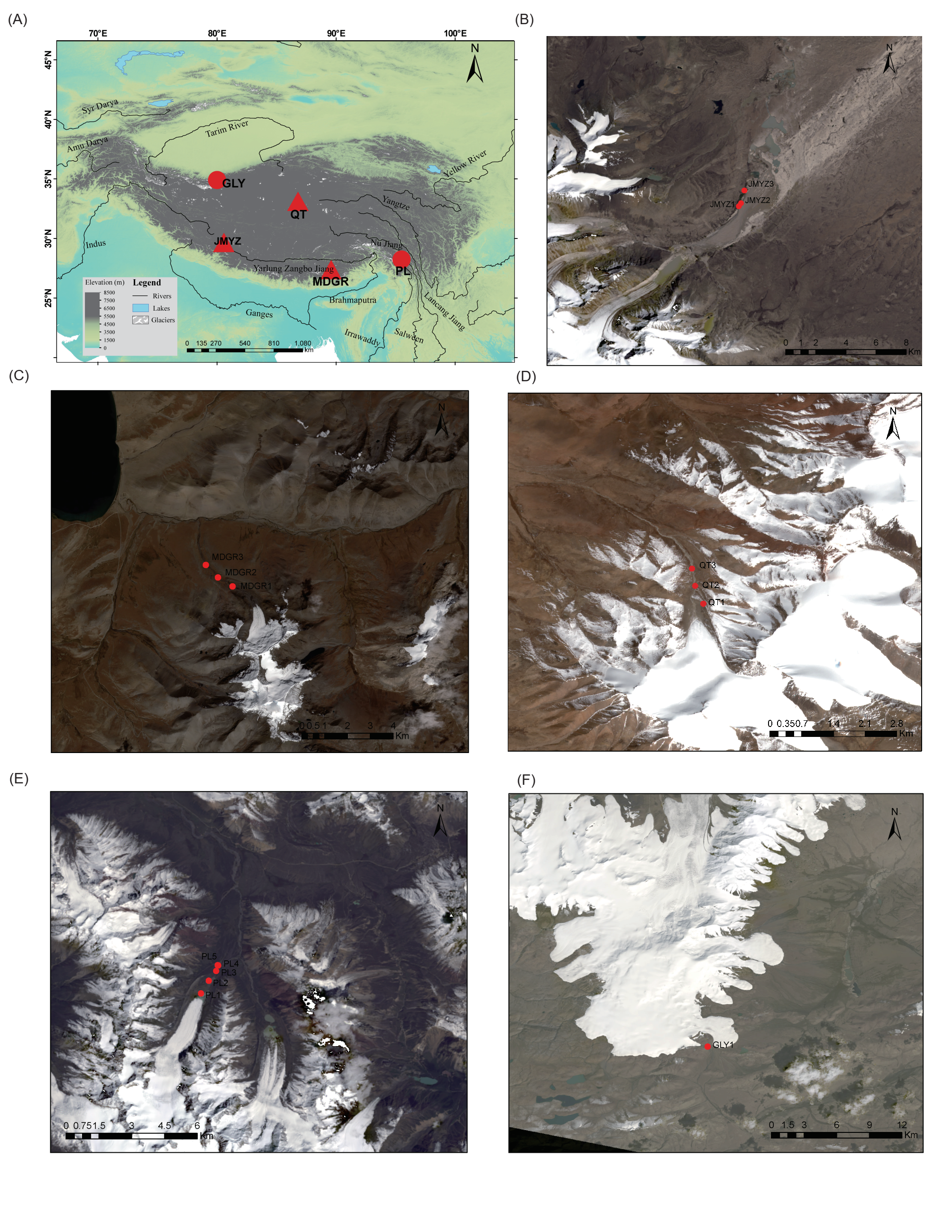


Figure S11. Map shows the soil sampling locations across the Tibetan Plateau. The overall map shows the location of glacier forelands; (A) Circle represents barren soils, and the triangle represents vegetated soils. (B) Vegetated soil samples collected in Jiemayangzong glacier; (C) Vegetated soil samples collected in Mengdagangri glacier; (D) Vegetated soil samples collected in Qiangtang NO.1 glacier; (E) Barren soil samples collected in Parlung glacier; (F) Barren soil samples collected in Guliya glacier. JMYZ, Jiemayangzong glacier; MDGR, Mengdagangri glacier; QT, Qiangtang NO.1 glacier; PL, Parlung glacier; GLY, Guliya glacier.
